# Calcium Nutrition and Extracellular Calcium Sensing: Relevance for the Pathogenesis of Osteoporosis, Cancer and Cardiovascular Diseases

**DOI:** 10.3390/nu5010302

**Published:** 2013-01-22

**Authors:** Meinrad Peterlik, Enikoe Kállay, Heide S. Cross

**Affiliations:** Department of Pathophysiology, Medical University of Vienna, Waehringer Guertel 18-20, A-1090 Vienna, Austria; E-Mails: enikoe.kallay@meduniwien.ac.at (E.K.); heide.cross@meduniwien.ac.at (H.S.C.)

**Keywords:** Calcium intake, calcium-sensing receptor, Wnt signaling, vitamin D, bone formation, colorectal cancer, breast cancer, hypertension, vasculature, cardiac functions

## Abstract

Through a systematic search in *Pubmed* for literature, on links between calcium malnutrition and risk of chronic diseases, we found the highest degree of evidence for osteoporosis, colorectal and breast cancer, as well as for hypertension, as the only major cardiovascular risk factor. Low calcium intake apparently has some impact also on cardiovascular events and disease outcome. Calcium malnutrition can causally be related to low activity of the extracellular calcium-sensing receptor (CaSR). This member of the family of 7-TM G-protein coupled receptors allows extracellular Ca^2+^ to function as a “first messenger” for various intracellular signaling cascades. Evidence demonstrates that Ca^2+^/CaSR signaling in functional linkage with vitamin D receptor (VDR)-activated pathways (i) promotes osteoblast differentiation and formation of mineralized bone; (ii) targets downstream effectors of the canonical and non-canonical Wnt pathway to inhibit proliferation and induce differentiation of colorectal cancer cells; (iii) evokes Ca^2+^ influx into breast cancer cells, thereby activating pro-apoptotic intracellular signaling. Furthermore, Ca^2+^/CaSR signaling opens Ca^2+^-sensitive K^+^ conductance channels in vascular endothelial cells, and also participates in IP_3_-dependent regulation of cytoplasmic Ca^2+^, the key intermediate of cardiomyocyte functions. Consequently, impairment of Ca^2+^/CaSR signaling may contribute to inadequate bone formation, tumor progression, hypertension, vascular calcification and, probably, cardiovascular disease.

## 1. Introduction

Calcium malnutrition can be linked to the pathogenesis of various chronic diseases (for review, [1,2,3,4]). Apart from intestinal dysfunctions causing impaired calcium absorption from the gut lumen, e.g., inflammatory bowel disease or lactase deficiency, today’s inadequate habitual calcium nutrition is the main reason for a low calcium status in millions of people worldwide [5]. 

Table 1 provides an update on levels of calcium intake in different population groups, in selected countries, in different geographical regions. As recommended dietary allowances (RDA) according to the 2011 report on Dietary Reference Intakes for Calcium and Vitamin D of the Institute of Medicine (USA) range from 1000 to 1300 mg calcium per day, depending on age and gender [6]. Data in Table 1 suggest that worldwide, many populations are at risk for a nutritional calcium deficit (for details, see [5]). However, one has to acknowledge that the interpretation of the evidence for calcium malnutrition is not always straightforward because of the great difference between studies with respect to design, methodology, and cohort size. However, the data collated in Table 1 confirm the long-standing assumption that, in many countries, certain population groups, such as the elderly, ingest significantly less calcium than the recommended amount, which is currently 1300 mg per day for this age group [6]. In addition, the extent of nutritional calcium insufficiency in schoolchildren, adolescents, and young women of childbearing age is of serious concern, particularly in South-East Asian countries, although daily calcium requirements in this region may be lower for ethnic reasons (see Section 4.9 in ref. [7]). 

**Table 1:** Actual and recommended dietary calcium intake in different population groups of selected countries.

Country	Age (years)	RDA ^a^ (mg/day)	Calcium intake (mg/day)		Study
			Gender		
			Male	Female	
***Europe***					
Austria	19–79	1000–1300	561 (±290) ^b^	576 (±309) ^b^	Kudlacek *et al.* [8]
Belgium	75–80	1300	748 (324–1166) ^e^	676 (287–1101) ^e^	Amorim Cruz *et al.* [9]
Denmark	70–75	1300		544 (127–1812) ^e^	Andersen *et al.* [10]
France	75–80	1300	620 (402–1010) ^e^	635 (428–944) ^e^	Amorim Cruz *et al.* [9]
Germany	18–79	1000–1300	1181 (902–1535) ^e^	1082 (849–1379) ^e^	Hintzpeter *et al.* [11]
Netherlands	75–80	1300	1036 (725–1447) ^e^	1010 (612–1616) ^e^	Amorim Cruz *et al.* [9]
Poland	70–75	1300		325 (86–851) ^e^	Andersen *et al.* [10]
***Middle East***					
Lebanon	10–16	1300	873 (793–952) ^d^	673 (595–750) ^d^	Salamoun *et al.* [12]
***America***					
Canada	18–35	1000		562 (0–2630) ^e^	Rubin *et al.* [13]
USA	31–50	1000	1118 (±25) ^b^	864 (±20) ^b^	Bailey *et al.* [14]
USA	>55	1300		611 (381–892) ^e^	Lappe *et al.* [15]
Brazil	16–20	1300	659 (596–721) ^d^	881 (730–1032) ^d^	Peters *et al.* [16]
***SE Asia***					
Bangladesh	16–40	1000		180 ^c^	Islam *et al.* [17]
Indonesia	18–40	1000		270 (239–302) ^d^	Green *et al.* [18]
Malaysia	18–40	1000		386 (353–420) ^d^	Green *et al.* [18]
China	>55	1300		485 (±253) ^b^	Kruger *et al.* [19]
Japan	65–75	1300		527 (±195) ^b^	Nakamura *et al.* [20]
***Oceania***					
Australia	20–94	1000–1300		643 (±340) ^b^	Pasco *et al.* [21]

^a^ RDA, recommended daily allowance according to the 2011 report on Dietary Reference Intakes for Calcium and Vitamin D of the Institute of Medicine, National Academy of Sciences USA [6]; ^b^ mean (±SD); ^c^ median (90% CI); ^d^ median (95% CI); ^e^ mean (range).

## 2. Calcium Malnutrition and Disease Incidence: Epidemiological Evidence

Over the years, a great number of observational and interventional studies indicated that chronic calcium malnutrition is associated with various diseases and pathologic conditions of unrelated etiology (for review, see [1,2,3,4]). These include osteoporosis and risk of falls and fractures, periodontal disease and age-related tooth loss, several types of cancer, hypertension, and cardiovascular disease. It is therefore not surprising that low calcium intake is associated with a significant increase in all-cause mortality as can be implied from a study by Rejnmark *et al.* [22]. These authors performed a patient level pooled analysis of eight major vitamin D trials and found a significant (*p* < 0.01) reduction of mortality when calcium was co-administered with vitamin D, while vitamin D alone had no effect. Furthermore, evidence mainly from animal studies suggests a link between low calcium status and disease incidence for autoimmune diseases such as inflammatory bowel disease and multiple sclerosis [23,24]. Although there is reason to believe that maintenance of adequate serum calcium levels is a prerequisite for normal function of the innate immune system [25,26], a link between a negative calcium balance and a specific infectious or chronic inflammatory disease has not yet been established in humans.

There is ample evidence that, in addition to calcium malnutrition, vitamin D insufficiency is a significant risk factor for a number of chronic diseases. These are in particular osteoporosis and related pathologies, as well as colorectal and breast cancer (for review, see [2]). Calcium and vitamin D reduce disease risks by activating different molecular and cellular mechanisms. Therefore, for efficient disease prevention, requirements for both calcium and vitamin D must be met. This is important in view of the high prevalence of combined calcium and vitamin D insufficiency worldwide [5]. 

### 2.1. Bone Diseases

#### 2.1.1. Calcium Deficiency and Rickets

Nutritional rickets is not only a sequel of vitamin D deficiency, but can also be caused by deficits in phosphate [27] or calcium [28]. There is evidence that in Nigeria, South Africa, Northern India, and Bangladesh a form of rickets occurs in older toddlers and children, which is only partially amenable to vitamin D, but can be fully resolved by calcium supplements alone [29,30]. The partial ineffectiveness of vitamin D could be due to the fact that two-thirds of the children with rickets had serum 25-(OH)D concentrations well above the rachitic range [30]. It has been suggested that, in this case, rickets is attributable to low dietary calcium intake, which is characteristic of cereal-based diets with limited variety and little access to dairy products [28]. According to De Lucia *et al.* [31], low dietary calcium intake after weaning is another reason for rickets with normal circulating 25-(OH)D, which is occasionally seen in the United States.

#### 2.1.2. Osteoporosis, Falls and Fractures

In 1998, Riggs *et al.* [32] had proposed a unitary model for involutional osteoporosis, in which, apart from hormonal imbalances, calcium malnutrition and calcium malabsorption are considered to be of great significance in the development of the disease in both genders. Consequently, nutrient and supplemental calcium has been widely used for the prevention and treatment of osteoporosis (e.g., [33,34,35,36,37,38,39]). A meta-analysis of randomized controlled trials by Boonen *et al.* [40] emphasized the need to co-administer calcium with vitamin D to significantly (*p* < 0.025) reduce the risk of hip fractures. Likewise, a patient level pooled analysis of seven major trials conducted in the USA and Europe, with fractures as endpoint [41] found that vitamin D alone is not effective in preventing fractures, but that calcium and vitamin D together significantly (*p* < 0.025) reduce the rate of total fractures, including hip and probably vertebral fractures as well. Tang and colleagues [42] provided further evidence that in people aged 50 years or older, daily doses of 1200 mg calcium in combination with 800 IU vitamin D have the best therapeutic effect to prevent fractures and osteoporotic bone loss. It is interesting to note that adult, though not elderly, African Americans experience less fragility fractures despite lower serum 25-(OH)D concentrations than whites in the same age range. Aloia [43] suggested that this seeming paradox can be explained by, among others, differences between African Americans and other populations in “calcium economy”. In other words, increased calcium absorption and superior calcium conservation might compensate, in African Americans, for the negative effect of vitamin D insufficiency on bone health.

A lower incidence of fractures could be partially due to the positive effect of calcium and vitamin D supplementation on neuro-musculoskeletal functions [44] leading to a reduction in the number of falls in the elderly [45]. Taken together, calcium in combination with vitamin D is the “gold standard” for treating older persons with decreased bone mineral density, increased propensity to falls, and osteoporotic fractures (for review, see [46]). 

### 2.2. Cancer

There is evidence that poor calcium nutrition is a significant risk factor for total cancer incidence [47], and, in particular, for colorectal and breast cancer. Low calcium intake may contribute to the development of renal, gastric, pancreatic, ovarian, endometrial, and lung cancer as well as multiple myeloma, though conclusive evidence is missing (for review, see [3]).

#### 2.2.1. Colorectal Cancer

Already in 1985, Garland *et al.* [48] had reported results of a 19 year prospective trial, which allowed the identification of low dietary calcium as an independent risk factor for colon cancer. Additional findings by Garland *et al.* [49] on an inverse association between nutritional calcium levels and colon cancer risk seem to indicate that the incidence of colon cancer can be reduced by approximately 70% if daily calcium intake is raised from 800 to 1400 mg. Since then, many other observational studies reported a strong association between incidence of colorectal cancer and low calcium intake: for example, Huncharek *et al.* [50] performed a meta-analysis of 60 epidemiological studies, and showed that high calcium intake, regardless of whether it is dietary or supplemental, had a significant protective effect against tumor development in the distal colon and rectum. Wu *et al.* [51] analyzed data from two large prospective trials, which had involved more than 80,000 women and 40,000 men. They reported a significant risk reduction for distal colon cancer for subjects whose dietary calcium intake was ≥1250 mg compared to those who ingested 500 mg or less. Cho *et al.* [52] analyzed pooled primary data from 10 cohort studies, in which more than half a million individuals were followed up for six to 16 years. They found a significant (*p* < 0.001 for trend) inverse relation between total calcium intake and colorectal cancer incidence. Comparable findings were reported by Jenab *et al.* [53], who had conducted a nested case-control study within the European Prospective Investigation into Cancer and Nutrition (EPIC) cohort of more than 520,000 participants from 10 western European countries.

It should be noted that the efficiency of calcium in reducing the risk of colorectal cancer depends very much on the vitamin D status of an individual*, *so that optimal prevention of the disease necessitates high intake levels of both vitamin D and calcium [52,53,54,55]. Notably, Fedirko *et al.* [56] reported a significant interaction (*p* < 0.01) between dietary calcium and vitamin D on reduction of colorectal cancer-related mortality in a nested case–control study within the EPIC cohort.

#### 2.2.2. Breast Cancer

From their studies on fat-induced carcinogenesis in mice, Lipkin and Newmark suggested that low dietary calcium could also promote breast cancer in humans [57].This notion gained strong support from multiple observations of an inverse relation between calcium intake and disease incidence in both pre-menopausal and post-menopausal women: Lin *et al.* [58] studied the effects of calcium intake from nutrient sources and supplements on breast cancer risk in a large cohort of premenopausal women. They found that higher intakes of total calcium were associated with a lower risk of premenopausal breast cancer (RR = 0.61; 95% CI: 0.40–0.92). Shin *et al.* [59] reported that in premenopausal women, dairy calcium (>800 mg/day *versus* ≤200 mg/day; RR = 0.69) had inverse associations with premenopausal breast cancer risk. McCullough *et al.* [60] analyzed data from nearly 70,000 postmenopausal women participating in the Cancer Prevention Study II Nutrition Cohort and found a moderately lower risk of breast cancer with intake of dietary calcium >1250 mg/day compared to <500 mg/day ((RR = 0.80, *p* [trend] = 0.02). This association was even stronger (RR = 0.67) in women with estrogen receptor-positive tumors. Consumption of dairy products was also inversely associated with risk (RR = 0.81; 95% CI, 0.69–0.95; *p* [trend] = 0.002) in this study. In a recent case-control study conducted among Chinese women by Zhang *et al.* [61], no significant association was found between dairy product intake and breast cancer risk, but a statistically significant inverse association of dietary calcium intake with breast cancer risk was observed with the adjusted OR (95% CI) of 0.35 (0.22–0.56) comparing the highest with the lowest quartile. Chen *et al.* [62] performed a meta-analysis of 15 studies on calcium intake and breast cancer risk: an inter-quantile comparison showing that high calcium consumption was associated with overall relative risk of 0.81 (95% CI = 0.72–0.90) adds to the evidence that calcium has a chemopreventive effect against breast cancer. 

Bérubé *et al.* [63] found that combined intake of calcium and vitamin D by pre-menopausal women was superior to separate intakes in reducing mammographic breast density, a surrogate marker of breast cancer incidence. This notion is valid also for breast cancer incidence as shown by Abbas *et al.* [64] in a population-based case-control study in Germany (for discussion, see [62]). 

#### 2.2.3. Prostate Cancer

Rather conflicting data have been reported with respect to the effect of calcium intake on the incidence of prostate cancer. In 1998, a study by Giovannucci *et al.* [65] found a positive association between calcium intake from food sources and supplements and risk of prostate cancer. Later, Gao *et al.* [66] analyzed 12 prospective studies published from 1966 to 2005. Dose-response analyses suggested that dairy product and calcium intakes were each positively associated with the risk of prostate cancer (*p* [trend] = 0.029 and 0.014, respectively). It was, however, not clear whether calcium intake itself was an independent risk factor for prostate cancer. This issue seemed to be clarified by Allen *et al.* [67], who analyzed data from almost the entire EPIC study cohort. They found that calcium from dairy products was positively associated with risk (*p* [trend] = 0.02), but not calcium from other foods. The situation elsewhere in the world appears to be less clear. Huncharek *et al.* [68] performed a meta-analysis of 45 observational studies, the majority of which were conducted in the United States. Results from 23 cohort studies and 26 case-control studies did not support an association between dairy product use and an increased risk of prostate cancer. While calcium data from cohort studies were heterogeneous and could not be used for the analyses, and respective data from case-control studies demonstrated no association with increased risk of prostate cancer. The results of this meta-analysis [68] should be seen with some caution because, as pointed out by the authors, “much of the reviewed literature predated the widespread use of PSA screening in the United States, and only few studies provided information on proportion of cases (controls) screened for prostate cancer and the stage distribution of cancer cases included in individual analyses”. When this information was available, somewhat different results were obtained: For example, Kristal *et al.* [69] examined nutritional risk factors for prostate cancer among nearly 10,000 participants in the Prostate Cancer Prevention Trial (United States and Canada, 1994–2003) and found that dietary calcium was positively associated (*p* [trend] = 0.165) with low-grade cancer but inversely associated (*p* [trend] = 0.034) with high-grade cancer. A preventive effect of high calcium intake against high-grade cancers was also found in a recent case-control study conducted by Williams *et al.* [70] among US veterans. 

### 2.3. Cardiovascular Disease

Calcium insufficiency has been correlated not only with cardiovascular risk factors, such as obesity, metabolic syndrome, diabetes mellitus type 2 and hypertension, but also with incident cardiovascular symptoms and stroke, as well as with greater mortality from chronic cardiovascular disease. However, the extent to which patients with high cardiovascular risk will benefit from calcium supplements is still under debate [71,72]. 

#### 2.3.1. Cardiovascular Risk Factors

*Obesity:* A clinical study of calcium intake with a skeletal endpoint showed a significant correlation between low calcium intake and increased body weight in women aged 30–80 years [73]. Findings from two studies that were conducted in the USA [73,74] suggested that high calcium intake significantly reduces the risk of obesity. In contrast, Puntus *et al.* [75] found no association between calcium intake and body mass index in a well controlled, large cohort study on healthy adult Austrians.

*Dyslipidemia and glucose intolerance *are, apart from hypertension and obesity, constituents of the so-called metabolic syndrome and of non-insulin-dependent diabetes mellitus. A link between low calcium and the metabolic syndrome and type 2 diabetes mellitus has been reported in observational studies (reviewed by [76]). The available evidence is limited because most observational studies were cross-sectional and did not adjust for important confounders.

*Hypertension: *Evidence from a large number of observational studies and randomized clinical trials indicates that low dietary calcium constitutes a significant risk factor for primary hypertension (for review, see [77]). Calcium appears to be particularly effective in reducing the age-related increase in blood pressure. Dobnig *et al.* [78] conducted a randomized, double-blind, multi-center study on the effect of daily high-dose calcium supplements in healthy, elderly adults and observed a substantial reduction of systolic and diastolic blood pressure after one year of treatment in individuals who were in the upper third of pre-study blood pressure values. No further improvement was seen with calcium *plus* vitamin D supplementation. It seems that, apart from hypertension, none of the classical risk factors of cardiovascular disease are significantly associated with low calcium intake. 

#### 2.3.2. Cardiovascular Disease: Incidence and Mortality

In 1999, Bostick *et al.* [79] had published results from a prospective cohort study of more than 30,000 Iowa women, suggesting that a higher intake of calcium is associated with reduced ischemic heart disease mortality in postmenopausal women. However, as mentioned previously, it is still a matter of debate whether higher intake of calcium also has a benefit on incident cardiovascular symptoms such as ischemic heart disease, myocardial infarction, and stroke [71,72]. For example, in a large cohort study of Japanese women and men, Umesawa *et al.* [80] found a significant inverse association of calcium intake with risk of stroke, particularly of the ischemic subtype, but did not detect any correlation with the risk of ischemic heart disease. The results of a 12 year follow-up of approximately 40,000 male health professionals in USA by Al-Delaimy *et al.* [81] suggested that neither dietary nor supplemental intakes of calcium were appreciably associated with the risk of ischemic heart disease among men. Lewis *et al.* [82] conducted a randomized placebo-controlled trial on the effect of calcium supplementation on the risk of atherosclerotic vascular disease in postmenopausal women. Patient-level analysis after a five year follow up period indicated that in patients with pre-existing atherosclerotic cardiovascular disease, calcium therapy reduced the risk of hospitalization and was associated with significantly fewer heart failure death events. 

Lewis *et al.* [82] interpreted their study that it “supplies compelling evidence that calcium supplementation does not have any adverse effect on myocardial function.” This statement refers to studies by Pentti *et al.* [83] and Bolland *et al.* [84,85], who had claimed that high calcium intake promotes calcification of coronary arteries and subsequent myocardial infarction. This notion, that was heavily debated in recent years, and particularly the studies by Bolland *et al.* [84,85] were heavily criticized (*cf.* [86,87]). In addition, Samelson *et al.* [88] most recently published the results of their prospective observational study on a cohort within the Framingham offspring study: The authors found no evidence that calcium intake from dietary sources, or from supplements, had any effect on coronary artery calcification. They also concluded from their study that there is no need “to modify current recommendations for calcium intake to protect skeletal health with respect to vascular calcification risk”. It should be noted that vascular calcifications develop secondary to vascular damage, which frequently results from vitamin D insufficiency and associated secondary hyperparathyroidism [89,90,91,92], whereas high calcium has been shown to prevent vascular damage and calcification through activation of the CaSR on vascular smooth muscle cells (for details, see Section 3.3.3).

## 3. Principles of Extracellular Calcium Sensing

**Figure 1:**
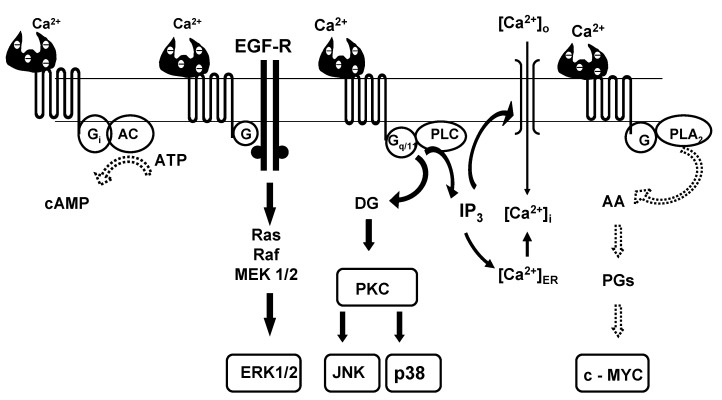
The extracellular calcium-sensing receptor (CaSR) senses, amplifies and transduces minute variations in [Ca^2+^]_o_ into various intracellular signaling pathways. Bold arrows indicate coupling to stimulatory G proteins, dotted arrows indicate coupling to inhibitory G proteins. Only cell-specific pathways relevant for the diseases mentioned in the text are shown (for more details on CaSR-activated pathways, see Magno *et al.* [119]).

Although the inverse relationship between dietary calcium intake and risk of multiple chronic diseases was known for years, it was not apparent how low levels of calcium intake could generate signals that were transduced to organs and cell systems distant from the intestinal lumen. Also, it is well known that the impact even of large variations in calcium intake on extracellular calcium concentrations [Ca^2+^]_o_ is attenuated by the systemic actions of calcium-regulating hormones, 1,25-dihydroxyvitamin D_3_ and parathyroid hormone (PTH). This allows physiological variations in [Ca^2+^]_o_ to occur only within a narrow range. How these can be translated into modulation of cellular functions became clear, when Brown and colleagues [93] cloned and characterized an extracellular calcium-sensing receptor (CaSR) from the bovine parathyroid gland. The CaSR, which was identified as a member of family C of G protein-coupled receptors, transduces small changes in [Ca^2+^]_o_, *i.e.*, in the range of 0.05 mM [94], along various intracellular signaling pathways (Figure 1).

Human cells that express this receptor include parathyroid chief, thyroid C and kidney cells [95], osteoblasts [96], gastric [97], and large intestinal epithelial cells [98], mammary gland [99], ovarian [100], prostate gland [101], pancreatic duct [102] and islet cells [103] as well as monocytes, macrophages, and dendritic cells [104]. The CaSR was also detected in arterial smooth muscle and endothelial cells [105]. Furthermore, in many regions of the brain, neuronal and glial cells express CaSR [106]. Expression of a functional CaSR allows extracellular Ca^2+^ to act as a “first messenger” for various intracellular signaling pathways that play an important role in control of cellular proliferation, differentiation, and function (for review, see [94,107,108]).

### 3.1. The Extracellular Calcium-Sensing Receptor (CaSR): General Properties and Function

The CaSR plays an important role in the maintenance of systemic calcium homeostasis mainly by modulating PTH secretion from the parathyroid gland [108]. It is known that, under physiological conditions, serum Ca^2+^ concentrations oscillate continuously within a narrow range. Amplitude and frequency of these oscillations are determined by the activity and cell-specific function of the CaSR on parathyroid gland chief cells. When, for example, due to reduced Ca^2+^ transfer from the intestine, the serum Ca^2+^ concentration falls to the lower physiological limit, this will change the activity of the parathyroid CaSR and thereby cause the release of PTH. When, as a consequence of PTH action on bone and kidney, serum Ca^2+^ reaches the upper physiological limit, activation of the CaSR leads to inhibition of PTH secretion, PTH gene expression, and parathyroid cell proliferation. Other effects of the CaSR that are relevant for systemic Ca^2+^ homeostasis include regulation of renal synthesis of 1,25-(OH)_2_D_3_ [109] and of calcitonin secretion from C cells in the thyroid gland [110]. The important role of the CaSR in systemic Ca^2+^ homeostasis is highlighted by results of a genome-wide association study, which showed that serum calcium in European and Asian Indian populations is significantly associated with single nucleotide polymorphisms near the CaSR gene, whereas no other locus reached genome-wide significance [111].

Expression of a functional CaSR allows cell-specific responses to physiological changes in [Ca^2+^] in the plasma and in other extracellular fluid compartments. Hence, the CaSR plays a key role not only in systemic Ca^2+^ homeostasis but also in local control of cellular functions, such as cartilage and bone formation [112,113,114,115], or growth limitation of normal and neoplastic cells [116,117]. While low calcium intake from diet can thus be linked to the development of osteoporosis and of various malignancies, involvement of the CaSR in the pathogenesis of other calcium insufficiency-related chronic diseases, such as hypertension and cardiovascular disease, is not yet fully understood.

### 3.2. The Extracellular Calcium-Sensing Receptor (CaSR): Molecular Properties

The human CaSR has a 612 amino acid extracellular domain, which is followed by 7 transmembrane helices built from 250 amino acids, and, finally, by a 216 residue carboxy terminal. The CaSR has a homodimeric structure with at least two high-affinity binding sites for Ca^2+^ in the extracellular domain of each monomer, which exhibit high positive cooperativity (Figure 1). This enables the CaSR to amplify the intensity of the otherwise minute signals from extracellular Ca^2+^ [108,118]. By coupling to stimulatory or inhibitory heterotrimeric G proteins the CaSR channels signals from extracellular fluid Ca^2+^ to various “second messenger”-generating systems, which include adenylate cyclase/cAMP, phospholipase A_2_ (PLA_2_)/arachidonic acid (AA) as well as phospholipase C (PLC)/diacylglycerol (DG) and inositoltrisphosphate (IP_3_). The latter is an important intermediate in CaSR-evoked Ca^2+^ influx through a non-selective cation channel [108] (Figure 1).

### 3.3. The Extracellular Calcium-Sensing Receptor (CaSR): Cell-Specific Actions

#### 3.3.1. Osteoblasts

**Figure 2:**
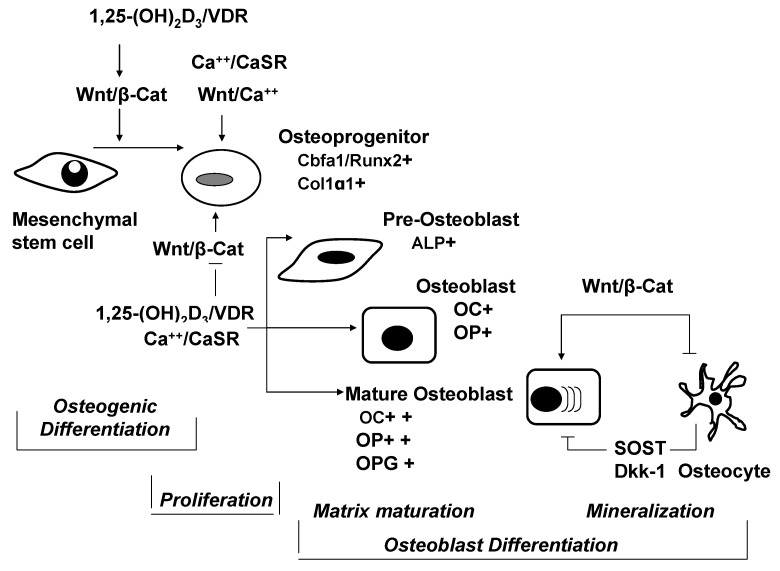
Cooperative signaling from Ca^2+^/CaSR and 1,25-(OH)_2_D_3_/VDR on Wnt pathways and formation of mineralized bone.

The concept of an important role of the CaSR in regulation of bone formation and mineralization as developed earlier [112,120], is fully supported by recent findings from a study in a CaSR knock-out mouse model [115]. As osteoblastic lineage cells are endowed with a functional CaSR (e.g., [121,122,123]), changes in [Ca^2+^]_o_ can be directly transduced into control of osteoblast proliferation, differentiation and function during any phase of mineralized bone formation (Figure 2). This process, in which the canonical Wnt/β-catenin signaling cascade plays a key role, [124] is coordinated by separate receptor-mediated effects of [Ca^2+^]_o_ and 1,25-dihydroxyvitamin D_3_ (1,25-(OH)_2_D_3_) [112,115,125]: As illustrated in Figure 2, activation of the Wnt/β-catenin pathway initiates “osteogenic differentiation” of pluripotent mesenchymal stem cells into osteoprogenitor cells, which characteristically express the osteoblast-specific transcription factor, Core-binding factor-1 (*Cbfa1*). Signaling from the CaSR in conjunction with the non-canonical Wnt5a/Ca^2+^ pathway [126] increases the expression of *Cbfa.* This causes osteoprogenitor cells to proliferate and to produce collagen [122]. Subsequently, signaling from 1,25(OH)_2_D_3_/VDR inhibits proliferation and matrix deposition and at the same time promotes osteoblast differentiation through phases of matrix maturation and mineralization [127]. Independent of vitamin D, activation of the CaSR increases the expression of osteoblast-specific differentiation markers, such as alkaline phosphatase (ALP), osteocalcin (OC) and osteopontin (OP). Finally, the CaSR plays an important role in orderly bone formation by regulating crystal growth and by preventing excessive mineralization [112].

#### 3.3.2. Cancer Cells

*Colorectal cancer:* The CaSR is expressed in normal human colonic epithelium, mainly in the upper half of the crypt, and is absent from the crypt base [128]. Thus, expression of the CaSR correlates with a [Ca^2+^]_o_ gradient that, according to Whitfield [129], decreases in the direction from top to base of the crypt. Pluripotent stem cells at the bottom of the crypt can thus proliferate without restraints from anti-mitotic signaling from the CaSR. Daughter cells, however, while migrating to the crypt top undergo differentiation under the influence of increasing CaSR expression and activity [130]. The assumption of an important role of the CaSR, in control of crypt cell homeostasis, is supported by findings in an intestinal-specific CaSR knockout mouse, which shows changes in crypt structure, expansion of the proliferative zone, and hyperproliferation of colonic epithelial cells [131]. There is suggestive evidence that the CaSR, on the luminal aspect of human colonocytes, reacts to ingestion of calcium-rich food by rapid activation of anti-mitogenic intracellular pathways [98].

The CaSR on human colon carcinoma cells has high sequence homology with the parathyroid CaSR [98,132], and is expressed as long as the cancer cells retain a certain degree of differentiation [128,133]. Epigenetic silencing of the CaSR could be one reason for reduced expression of the receptor in more advanced cancers and in nearly all lymph node metastases [134]. From the evidence that expression of the CaSR is highest in well differentiated cancers and declines during tumor progression [128,133], it can be implied that calcium acting via the CaSR is a chemopreventive rather than a chemotherapeutic agent.

**Figure 3:**
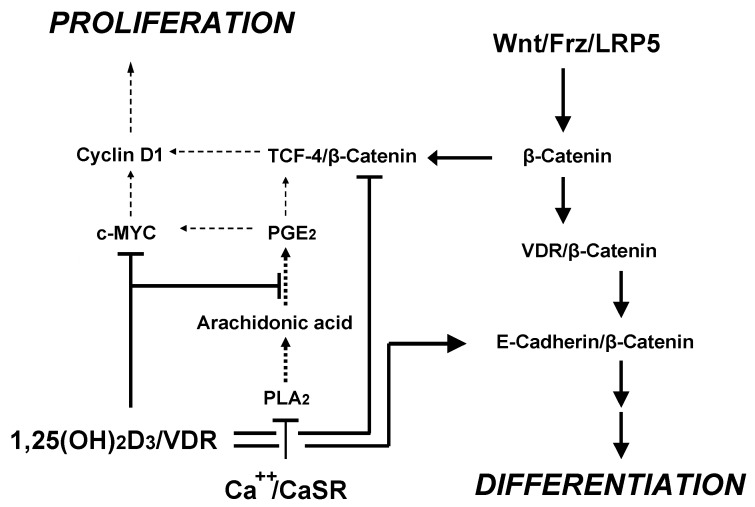
Cooperative signaling from Ca^2+^/CaSR and 1,25-(OH)_2_D_3_/VDR on Wnt pathways inhibits proliferation and promotes differentiation of colonocytes and of colorectal cancer cells.

The sequence of events downstream of CaSR activation that actually link CaSR to cell cycle control in colon carcinoma cells starts with inhibition of phospholipase A_2_ activity [135] (Figure 1 and Figure 3), which would reduce the amount of arachidonic acid available for the synthesis of proliferation-stimulating prostaglandins. Subsequent down-regulation of c-*myc* proto-oncogene expression [98], activation of the cyclin-dependent kinase inhibitor p21 [136] and inhibition of cyclin D1 finally leads to cell cycle arrest at the G_1_/S-phase transition. CaSR-activated pro-differentiating signaling in colonocytes involves inhibition of the Wnt/β-catenin pathway by down-regulation of the T-cell transcription factor (TCF)-4 with subsequent induction of E-cadherin expression [137,138,139]. Interestingly, part of the anti-proliferative action of 1,25(OH)_2_D_3_ has been traced to a VDR-mediated, negative effect on TCF-4 [137,140] (Figure 3). These findings illustrate that Ca^2+^ and vitamin D independently inhibit colorectal cancer cell proliferation. Therefore, a combination of both agents is well suited to reduce the risk of colorectal cancer. 

*Breast cancer: *A role for a functional CaSR in breast cancer can be inferred from the fact that in premenopausal women serum calcium levels vary inversely with breast cancer risk in a concentration-dependent manner [141]. Both normal and malignant mammary gland epithelial cells are endowed with the CaSR [99]. However, little is known of the role played by the CaSR in mediating changes in ambient Ca^2+^ to regulate cellular growth in mammary gland cells. In MCF-7 breast cancer cells, activation of the CaSR is transduced via the PI-PLC pathway into generation of IP_3_, which not only causes the release of Ca^2+^ from endoplasmic reticulum, but also evokes Ca^2+^ influx across the plasma membrane through non-selective cation channels (Figure 1; for details, [108,142]). The rise in intracellular Ca^2+^ may conceivably activate pro-apoptotic intracellular signaling [143], particularly through involvement of different plasma membrane calcium ATPase isoforms [144]. In addition, the CaSR can inhibit breast cancer cell growth through its functional linkage with the tumor-suppressor BRCA1: Promkan *et al.* [145] reported that BRCA1 up-regulates the expression of the CaSR and, at the same time, functions through the receptor in suppressing the expression of survivin, an anti-apoptotic protein that is present in most cancer cells. 

An apparent cross-talk between Ca^2+^/CaSR and 1,25(OH)_2_D_3_/VDR signaling [146] on cytosolic Ca^2+^ may explain, at least in part, how vitamin D and calcium together efficiently inhibit mammary gland cell growth *in vivo *(*cf.* Section 2.2.2).

Activation of the CaSR could have dual effects on disease outcome: On the one hand, activation of the CaSR in human MCF-7 breast cancer cells by extracellular calcium concentrations that are detected in bone at tumor metastasis, enhances expression of the estrogen receptor-alpha [147]. An improved estrogen receptor status is considered a good prognostic factor. On the other hand, the CaSR has also been shown to stimulate synthesis and secretion of parathyroid hormone-related peptide, which is thought to play a key role in osteolytic metastases [148].

*Prostate cancer:* The CaSR is expressed on human prostate cancer cell lines [101,149,150]. Unlike colorectal and breast cancer cells, prostate cancer cells respond to high calcium with increased proliferation. This may be due to transactivation by the CaSR of the epidermal growth factor receptor (EGFR) and subsequent activation of ERK1/2 (*cf. *Figure 1) and release of PTHrP [101,149]. This can explain why prostate cancers expressing high levels of the CaSR are more likely to metastasize to bone than those poorly expressing the CaSR. Stimulation of PTHrP release from bone metastatic prostate cancer cells induces osteoclastic bone resorption, whereby the Ca^2+^ concentration in the resorptive lacunae of osteoclasts rises to eight to 40 mM [65]. High calcium, acting through the CaSR, causes malignant prostate cells to proliferate and to release even more PTHrP, leading to increased osteolytic activity and enhanced survival of metastatic cells in the bony microenvironment [65]. The relevance of CaSR expression for metastases development, and for disease outcome, is underscored by the recent findings of Shui *et al.* [151] that common genetic variants of the CaSR are significantly associated with prostate cancer mortality. 

#### 3.3.3. Cardiac, Vascular, Renal Juxtaglomerular and Epithelial Cells

Because current literature on expression of the CaSR in human arterial and cardiac cells is rather limited, inference on the function of the receptor in the human cardiovascular system has to be made from the wealth of information on the expression and function of the CaSR in the vasculature (heart and blood vessels and associated structures) of rodents and other experimental animals. In addition, a substantial role of the CaSR in maintaining normal blood pressure and cardiovascular functions is supported by studies on the effect of so-called calcimimetics, *i.e.*, synthetic allosteric activators of the CaSR, in various animal models [152] and patients with chronic kidney disease [153]. It is now very clear that the CaSR is part of an intricate network of calcium channels, pumps, and exchangers, which is involved in the control of intracellular calcium concentration ([Ca^2+^]_i_) and thereby in modulation of cardiovascular functions [154,155]. 

*Cardiomyocytes:* The CaSR is functionally expressed on rat neonatal and adult cardiomyocytes [154,156,157]. Upon activation by [Ca^2+^]_o_ the receptor transduces signals preferentially along the PLC pathway causing intracellular accumulation of IP_3_ (*cf. *Figure 1) and, in turn, a rise in [Ca^2+^]_i_, the classic “second messenger” in cardiac physiology. Thus, the CaSR has been implicated in regulating cardiac development, function, and homeostasis [154]. Apart from regulating [Ca^2+^]_i_, the CaSR may be involved in excitation-contraction coupling through a unique feedback mechanism. Hofer *et al.* [158] observed that intracellular signaling events can produce changes in [Ca^2+^]_o_ that are detected by the CaSR on cells in close proximity. According to Tfelt-Hansen *et al.* [154], the CaSR on cardiomyocytes may therefore change its activity with every heart contraction.

*Endothelial and smooth muscle cells* of human arteries express a functional CaSR [105]. The receptor was detected also in endothelial cells from rat mesenteric and porcine coronary arteries [155], and in rat aortic smooth muscle cells [152]. Indeed, the CaSR is functionally expressed on all cells of the vascular wall-adventitia, fibroblast, vascular smooth muscle cells (VSMC), and endothelium. It is closely involved in the regulation of vascular tone and arterial blood pressure (for review, see [159]). In a hypothetical model proposed by Weston *et al.* [155] the CaSR plays a key role in myo-endothelial coupling in the vasculature. Activation of the CaSR induces endothelium-dependent hyperpolarization in neighboring myocytes. In short, activation of the endothelial CaSR is associated with the opening of a Ca^2+^-sensitive K^+^ conductance channel. K^+^ released into the intercellular space is taken up by the myocyte Na^+^/K^+^-ATPase. This contributes to a reversal of spasminogen-induced depolarization of vascular myocytes and, consequently, to vascular relaxation. This can explain, at least in part, the well documented hypotensive effect of dietary Ca^2+^ (see section 2.3.1). 

The CaSR has also been shown to be involved in vascular repair and maintenance of vascular integrity [159]. Normal expression of the receptor on VSMC seems to be important for prevention of vascular calcification, since calcification is associated with reduced expression of the CaSR in blood vessels [160]: large and small arteries of normal subjects and of patients with advanced chronic kidney disease express the CaSR, although the expression is lower in epigastric arteries of patients with advanced renal impairment, compared with healthy transplant donors. Analysis of epigastric arteries, taken from chronic kidney disease patients, showed that a decrease in CaSR expression was accompanied by progressive calcification of VSMC areas [105]. Moreover, there is increasing evidence from *in vitro* and *in vivo* studies that calcimimetic compounds upregulate the CaSR in vascular cells and thereby attenuate vascular mineralization associated with reduced kidney function (for review, see [153]).

*Juxtaglomerular cells:* According to Atchison and Beierwaltes [161], signaling from Ca^2+^/CaSR in juxtaglomerular cells inhibits a calcium-inhibitable isoform of adenylate cyclase (*cf. *Figure 1), which is associated with formation of renin. Reduced activity of the renin-angiotensin II-aldosterone system could partially account for a CaSR mediated hypotensive effect of dietary and supplemental calcium.

*Renal epithelial cells: *Activation of the basolateral CaSR in the thick ascending limb of Henle’s (TAL) mediates the anti-hypertensive effect of elevated extracellular calcium in two ways: (i) CaSR signaling via PKC results in up-regulation of COX-2 and enhanced synthesis of the natriuretic prostaglandin E_2_ [162,163]; (ii) by activation of PLA_2_, CaSR signaling targets apical electrolyte transporters and channels participating in the control of electrolyte reabsorption from the lumen [164]. The importance of the CaSR in this process has been proven by identification of activating mutations in the CaSR gene, which cause Bartter syndrome type 5, a human disease characterized by excessive wasting of NaCl and other electrolytes [165]. Taken together, signaling from the basolateral CaSR in the TAL induces natriuresis and thereby causes reduction of plasma volume. This can partially explain the blood pressure-lowering effect of elevated extracellular calcium

## 4. Conclusions

There is no doubt that the CaSR plays an important role in local control of cellular homeostasis and function in many tissues and organs. Impaired or malfunctioning signaling from Ca^2+^/CaSR may therefore contribute to the adverse health effects of low habitual calcium intake. Because of the synergistic action of Ca^2+^/CaSR-activated and vitamin D receptor-mediated signaling cascades, one should bear in mind that, for optimal health outcome, restoration of both an adequate calcium and vitamin D status is necessary. There is evidence that efficient disease prevention does not require intake of more calcium and vitamin D than currently recommended for maintaining optimal bone health [166]. 
